# Deep learning-based system for prediction of work at height in construction site

**DOI:** 10.1016/j.heliyon.2025.e41779

**Published:** 2025-01-17

**Authors:** Ibrahim Karatas

**Affiliations:** Osmaniye Korkut Ata University, Faculty of Engineering and Natural Sciences, Department of Civil Engineering, Osmaniye, Turkey

**Keywords:** Construction safety, Fall from height, Deep learning, Construction management

## Abstract

Falling from height (FFH) is a major cause of injuries and fatalities on construction sites. Research has emphasized the role of technological advances in managing FFH safety risks. In this investigation, the objective is to forecast if a laborer is operating at an elevated position by utilizing an accelerometer, gyroscope, and pressure information through the application of deep-learning techniques. The study involved analyzing worker data to quickly implement safety measures for working at heights. A total of 45 analyses were conducted using DNN, CNN, and LSTM deep-learning models, with 5 different window sizes and 3 different overlap rates. The analysis revealed that the DNN model, utilizing a 1-s window size and a 75 % overlap rate, attained an accuracy of 94.6 % with a loss of 0.1445. Conversely, the CNN model, employing a 5-s window size and a 75 % overlap rate, demonstrated an accuracy of 94.9 % with a loss of 0.1696. The results of this study address information gaps by efficiently predicting workers' working conditions at heights without the need for complex calculations. By implementing this method at construction sites, it is expected to reduce the risk of FFH and align occupational health and safety practices with technological advancements.

## 1 Introduction

Construction sites have always been known as one of the most hazardous industries due to the unique nature of outdoor operations, working at heights, complex facilities and equipment on site, and employee attitudes and behaviors towards safety [1,2]. Construction projects not only have hazardous working environments but also subject workers to intense physical exertion and serious safety and health risks, all leading to an increase in fatal and non-fatal accidents [3]. Globally, the construction sector employs approximately 29 % of all industrial workers, yet it has the highest rate of occupational accidents among all sectors at 40 % [4]. In the last 20 years, over 28,000 workers have died in the construction industry, with approximately 200,000 non-fatal injuries reported annually [5]. The most common accidents in the construction industry include falls from heights (FFH), collisions with objects, electrocution, and being trapped [6]. Moreover, there has been an increase in industrial accidents in South Korea, with accidents at construction sites making up over one-third of all industrial accidents. Falls account for 47.7 %–52.1 % of the total number of fatalities in the construction industry [6]. In addition to the United States and South Korea, other countries such as Australia, China, and Turkey have experienced significant economic and human losses due to FFH [2,7,8]. Given the presence of elevated work areas such as ladders, scaffolding, and roofs, construction workers in such environments are at a heightened risk of injury and fatality. Previous studies have emphasized the significance of proactive and preventive measures, including hazard identification, control, safety training, and the prompt recognition of fall hazards on construction sites, as crucial in mitigating these risks [2,6,8,9]. In the context of FFH accidents, it is essential to identify and address the fall hazards present at construction sites and communicate these situations to experts in order to minimize such incidents. While safety training programs have been implemented to mitigate risks, they have only been partially effective in reducing the number of falls at construction sites [8,10]. Furthermore, safety equipment like seat belts has been employed to mitigate the risk of FFHs. Nonetheless, although conventional measures such as seat belts are effective in minimizing the impact of risks, documented incidents of FFH reveal that traditional methods are inadequate in addressing the diverse fall hazards present in today's dynamic work settings [11]. Hence, it is imperative to employ cutting-edge technologies in order to mitigate these incidents and enhance the well-being and safety of laborers [8,[12], [13], [14], [15]].

Numerous new technologies have been developed and researched in the literature to identify and mitigate ongoing fall accidents at construction sites. These include integrating building information modeling (BIM) systems with safety performance to improve hazard detection and communication [1,3,16,17], using a camera or closed-circuit television system to monitor construction activities [[18], [19], [20]] and employing wearable technologies to recognize unsafe behavioral activities and improve safety management [2,3,[21], [22], [23]]. Effective occupational health and safety measures rely on three fundamental pillars: prediction, prevention, and mitigation. These pillars form the basis for enhancing safety risk management procedures, judgments, and results through the utilization of suitable technologies [24]. Thus, the primary objective of this research is to propose a methodology for forecasting the occurrence of elevated work among workers using data collected through sensors. This approach involves implementing preventive measures based on the generated forecasts, ultimately leading to a reduction in incidents of FFH.

Contemporary technological advancements possess the capability to adequately mitigate the hazard of falling from elevated surfaces. Appropriate utilization of technological tools in preempting fall hazards within the realm of occupational health and safety in construction environments has the potential to enhance the accuracy of risk anticipation [24]. Conversely, the significant expenses associated with the hardware and software components of contemporary technologies, the necessity of providing training for staff members on the utilization of such technologies, and the impact of wearable devices on the limited movement of employees are identified as obstacles in this context [25,26]. However, in spite of these obstacles, the utilization of emerging technologies presents an opportunity for a proactive strategy in the anticipation and management of fall hazards [26].

In the realm of occupational health and safety, extensive research has been carried out regarding the utilization of novel technologies for the anticipation and mitigation of fall incidents from elevated positions. Fang et al. (2019) introduced an automated computer vision methodology employing Mask Region Based Convolutional Neural Network (R-CNN) for the identification of hazards among construction site personnel. The study revealed recall and precision rates of 90 % and 75 % respectively for hazard identification using this particular approach. The authors suggested that the computer vision system could be implemented by construction site supervisors to autonomously identify unsafe actions and offer guidance to individuals regarding their risk of experiencing an FFH [27]. Anjum et al. (2022) introduced a deep learning-based method for assessing height. This method uses a single known value in an image to measure the working height of workers, monitor compliance with safety regulations, and ensure worker safety. The study analyzed over 300 images for binary classification (safe and unsafe) and achieved an overall accuracy of 85.33 % [6]. In a study concerning the monitoring of workers, Hong et al. (2023) introduced a framework aimed at categorizing the predetermined safety actions of scaffold workers and assessing the fulfillment of each safety action, with the purpose of enhancing safety management in response to the challenges associated with manual observations. To achieve this objective, they developed a Convolutional Neural Network (CNN) model utilizing Gramian angular fields (GAFs) to categorize adherence to safety protocols among 35 scaffold workers through the utilization of five Inertial Measurement Unit (IMU) sensors. The outcomes of the model indicate that the proposed approach has the potential to automatically discern whether workers are adhering to safety regulations or not [28]. Automatic detection of fall hazards using IMU sensors, including accelerometer and gyroscope data, has been identified as an effective approach to monitoring fall hazards from height on construction sites [29]. In this way, it is possible to take timely preventive measures against dangerous situations. Additionally, these wearable sensors can help prevent FFH by enabling advanced precautions.

Choo et al. (2023) have proposed a system that utilizes wearable sensors to identify workers operating at heights and determine the anchoring status of safety hooks in order to prevent falls. The IMU sensor collects data, which is then processed using machine learning algorithms to assess the status of safety hooks for workers at heights. Despite previous efforts to identify unsafe working conditions and behaviors at heights, researchers have encountered challenges due to the complexity of tasks and dynamic working conditions, which have hindered the establishment of precise methodologies for effective and timely detection. The evaluation of the constructed model was conducted through single-subject cross-validation (LOSOCV) in order to accommodate various new workers and working environments. As per the findings, the system for identifying work at height demonstrated an accuracy of 96 %, whereas the system for detecting safety hook attachments exhibited 86 % accuracy [2]. In addition, in the work on the height detection system by Choo et al. (2023), a rule-based height estimation was made by applying the Kalman Filter and Complementary Filter algorithms to the collected data. In addition, it was determined whether the worker was only working at height or not.

In the evaluation of construction studies, there is a notable interest in leveraging technology to enhance occupational health and safety practices. It is evident that the adoption of technology at construction sites substantially reduces accidents and injuries, thereby cultivating a safer work environment. Specifically, there is a heightened focus on addressing FFH incidents for the overall well-being of workers. Extensive research has delved into identifying fall hazards in the construction industry. Specifically, computer vision-based studies have been employed for this purpose. However, the analysis process can be time-consuming and demanding due to the high processing requirements of image processing stages. Furthermore, existing studies have primarily focused on identifying and predicting two scenarios: whether workers are operating at elevated heights or not. The dataset employed in this research comprises the information gathered by Choo et al. (2023) in their study. In contrast to their work, the objective is to develop a deep learning-based prediction model specifically for detecting work at heights. Furthermore, the aim is to predict not only work at heights in general but also work at heights on the ground, on ladders, on mobile scaffolds, and horse scaffolds separately. In the context of creating deep learning models, it is imperative that the data obtained from sensors adheres to specific window sizes and overlap ratios. To achieve this, the sliding window technique is commonly employed. This methodology is frequently employed in the realm of human activity recognition challenges. It is specifically utilized in the initial phases of data segmentation to process unrefined data for subsequent modeling purposes. The popularity of this method stems from its simplicity of implementation and its exemption from preprocessing requirements. Particularly advantageous for applications requiring instantaneous responses, this method presents an optimal solution. The process involves segmenting the incoming sensor data into windows of a consistent size, with no intervals between them [30]. In addition to the dimensions of the window, the percentage of overlap plays a significant role in the context of activity recognition tasks when windows traverse one another. Prior research has employed window durations varying from 0.1 to 8 s along with diverse overlap percentages during the process of segmenting data [[30], [31], [32], [33], [34], [35], [36], [37], [38]]. One of the aims of this study was to determine the optimal window size and overlap ratio for predicting the analyzed data. The window sizes tested were 1s, 2s, 3s, 4s, and 5s, with overlap ratios of 25 %, 50 %, and 75 %. This was necessary to divide the data collected at 25Hz fully and achieve the highest prediction success.

The main contributions of our study are as follows.

• By utilizing data from accelerometers, gyroscopes, and pressure sensors gathered from individuals in a workplace setting, one can potentially infer the worker's occupational conditions and identify the necessary safety measures for tasks performed at elevated locations.
• In exploring this study, it has been aimed to ascertain the most precise model through a comparative analysis of various deep-learning approaches for predicting working at height situations.
• Analyzing various window sizes and overlap ratios for analysis, along with different deep learning methods, to determine the optimal window size and overlap ratios for the most accurate model.

### 1.1 Knowledge gaps

In academic literature, researchers have utilized accelerometers and gyroscopes to assess workers' movements and their impact on efficiency at construction sites. However, there is limited research on the use of these sensors in studying occupational health and safety, particularly in relation to falls from height. Furthermore, image-processing techniques have also been employed in studies pertaining to occupational health and safety. This study emphasizes the effectiveness of utilizing kinematic-based sensors to navigate the complexities of construction site environments. These sensors are both lightweight and wireless, which enhances their practicality for real-world applications. Also, in contrast to most similar research that relies on traditional machine learning techniques, our approach employs advanced deep learning methods to improve accuracy rates significantly. The proposed deep learning framework allows for rapid predictions without necessitating complex calculations for operations at heights, and it also facilitates swift preventive measures against falls from heights (FFH). A range of deep learning models was evaluated to identify the one with the highest accuracy. Furthermore, this research explores various options to establish the optimal window size and overlap ratio during the data segmentation phase, which is crucial for determining the best data preprocessing techniques before model development. These investigations set this study apart from previous research in height level estimation.

## 2 Research methodology

**Fig. 1:**
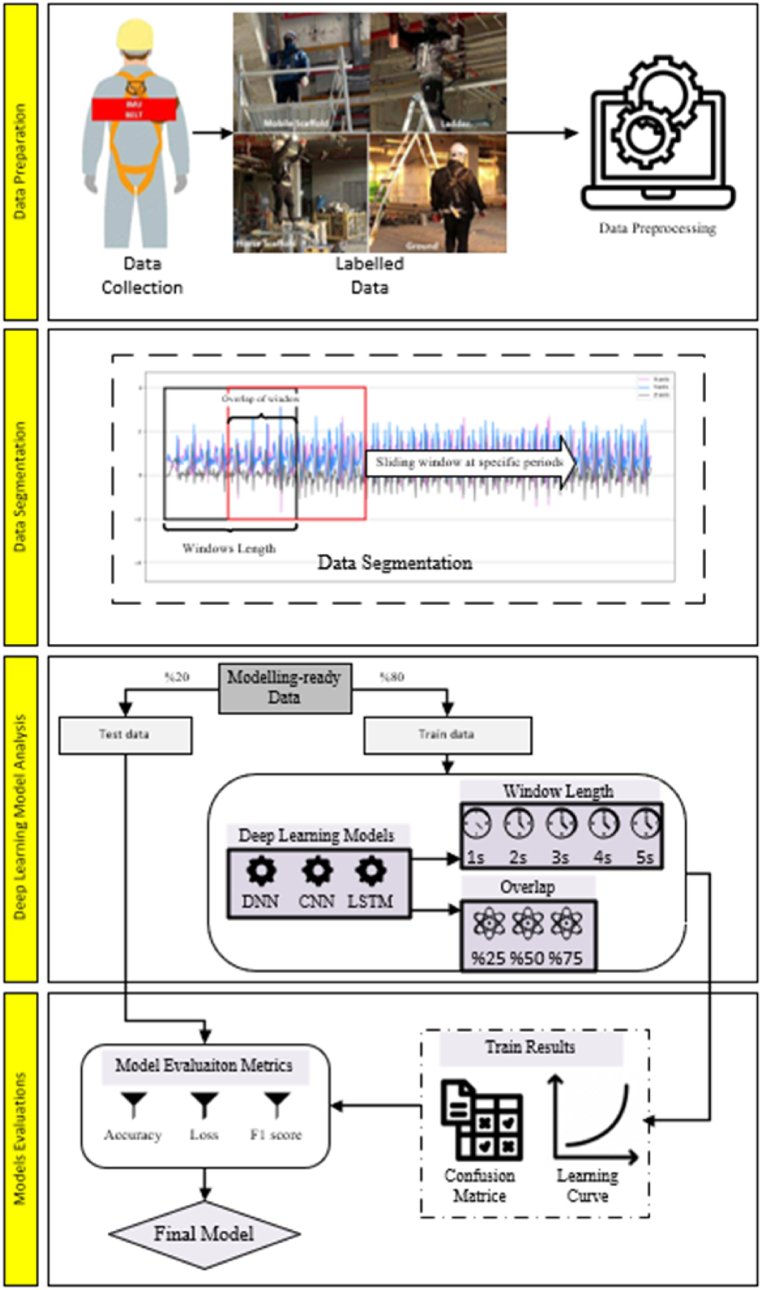
Research Methodology flowchart.

This research aims to predict the height levels of construction workers by analyzing data collected from accelerometers, gyroscopes, and barometers using advanced deep-learning techniques. Additionally, it seeks to identify the approach that provides the highest accuracy in these predictions by evaluating various window sizes and overlap ratios within the deep learning methods employed for height level estimation. The objective is to accurately forecast the working height levels of workers, while also exploring the feasibility of a framework designed to monitor their safety. This framework will investigate whether the working heights of construction workers can be effectively estimated through the combination of motion sensor data and barometric data. This study is grounded in two primary hypotheses: (1) to explore whether distinctive features derived from motion data—such as accelerometer, gyroscope, and barometric readings—can be effectively used to estimate a worker's height level, and (2) to determine the optimal window size and overlap ratio for analysis using a deep learning method that achieves the highest accuracy in estimating the height level of construction workers. A schematic representation of the research methodology, aligned with this framework, is illustrated in Fig. 1.

The initial step involves preparing the data obtained from the literature for the model. The data was labeled according to the method described in Choo et al. (2023) [2]. Following data preprocessing and segmentation, the dataset has been split into training and test sets employing 5 distinct window sizes and 3 different overlap rates. The performance of the training data has been compared with that of the test data to assess prediction accuracy. Subsequently, 5 different deep learning models have been applied to analyze the data, and model evaluation metrics have been utilized to identify the most suitable model.

A. Data Preparation and Data Segmentation

**Fig. 2:**
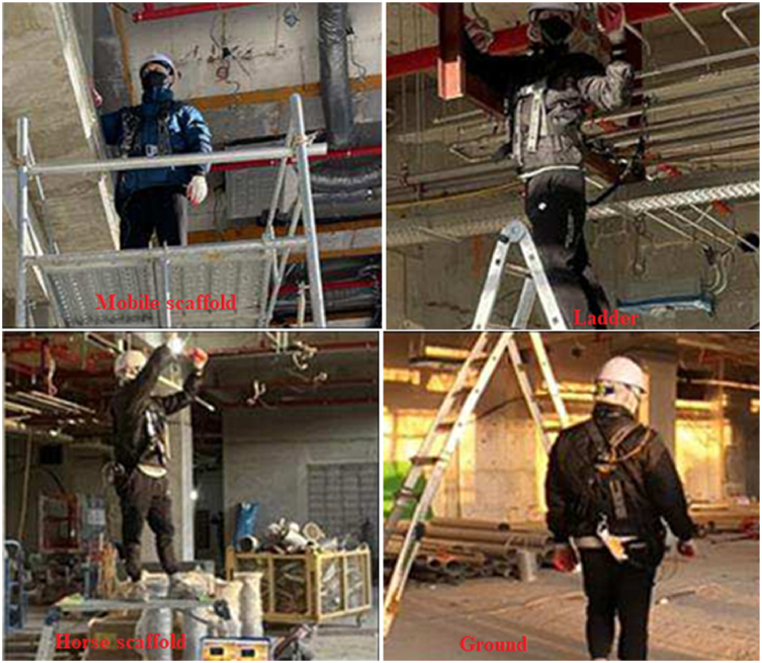
Types of scaffolding for which data was collected [2].

The study utilized data from Choo et al. (2023) [2] and involved 20 workers in a construction site setting. The data was gathered from sensors located at three different positions: base, belt, and hook. However, only the data from the belt sensors was used in this study to predict working at heights. The area labeled as the "base" refers to the sensor mounted on the floor, while the "hook" designates the sensor attached to the end of the worker's safety hook. The term "belt" indicates the sensor positioned on the safety belt worn by the worker. Given that the worker consistently wears a seat belt, the data gathered from the sensor affixed to the belt is utilized in this research. Each sensor captured accelerometer, gyroscope, and barometer data. In this study, data consisting of atmospheric pressure signals from the barometer, as well as acceleration and gyroscopic signals from the Inertial Measurement Unit (IMU) sensor, were recorded at a frequency of 8 Hz. The descriptive statistics for this dataset, comprising a total of 368647 entries, are presented in Table 1. This study aims to assess the utilization of scaffolds by workers during their work activities. Subsequently, based on this assessment, an evaluation of their work performance at elevated heights has been conducted. The method employed in the research article facilitated the identification of the specific type of scaffold (such as mobile scaffold, horse scaffold, ladder, or ground) on which the monitored workers were operating, as illustrated in Fig. 2. The collected data was subsequently labeled. Following an initial analysis, outlier values were eliminated using the median absolute deviation (MAD) method.

**Table 1:** Descriptive statistics for the dataset.

	Pressure	Acc_x	Acc_y	Acc_z	Gyr_x	Gyr_y	Gyr_z
**Mean**	1017,10	1186	2726	9142	0,038	−0,056	−0,017
**Std**	5,33	2260	1486	0,927	0,203	0,269	0,506
**Min**	1008,88	−19,321	−13,195	−17,339	−7341	−12,067	−4889
**25 %**	1010,92	0,464	1853	8867	−0,043	−0,161	−0,205
**50 %**	1017,88	1714	2833	9251	0,038	−0,056	−0,018
**75 %**	1020,69	2623	3651	9596	0,117	0,051	0,167
**Max**	1026,39	36,982	14,161	24,309	1847	5942	5118

**Fig. 3:**
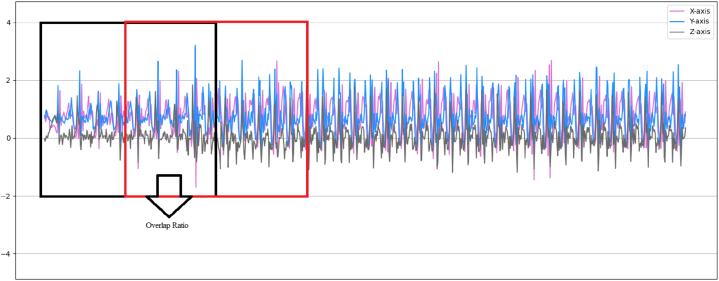
Data segmentation process.

Following the collection of raw data, data segmentation was conducted to prepare it for the model. This process entailed dividing the data into windows of specific lengths and shifting them over each other with a predetermined overlap rate until the final data was ready for the model. Window sizes ranging from 0.1 to 10 s, as depicted in Fig. 3, have been identified as appropriate for activity recognition based on earlier investigations. The overlap rate among pennies in past research typically ranged from 25 % to 75 % [30,[39], [40], [41]]. In this research, data was gathered at a frequency of 8 Hz, with overlap rates of 25 %, 50 %, and 75 % being chosen. Consequently, window sizes of 1 s, 2 s, 3 s, 4 s, and 5 s were determined for complete separation of the data. Following this process, the resulting segmented data was prepared for model input. After segmentation, the data was split into 80 % for training and 20 % for testing. Three deep learning techniques (DNN, CNN, LSTM) were employed for activity classification in this study.

B. Deep Learning Models

In the domain of machine learning models, the process of statistical feature extraction plays a pivotal role in the examination phase. Nevertheless, this procedure may consume considerable time and resources. Conversely, deep learning techniques present a more effective strategy for feature extraction. These techniques scrutinize motion data instead of statistical characteristics, leading to a substantial decrease in the time and resources necessary for examination. Through the utilization of deep learning techniques, organizations and educational entities can optimize their examination procedures and attain more precise outcomes in a shorter time frame.

### 2.1 Deep neural network (DNN)

**Fig. 4:**
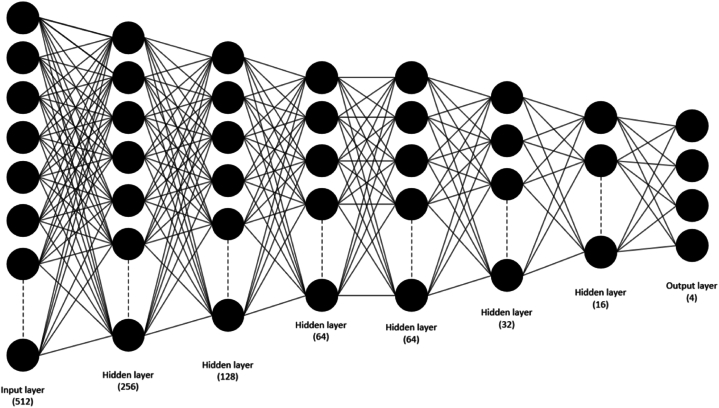
DNN model architecture.

Neural networks usually consist of a restricted number of hidden layers. In contrast, a Deep Neural Network (DNN) is essentially a standard neural network with added "depth". The depth of a neural network is defined by the number of hidden layers positioned between the input and output layers. DNNs are more adept at processing large datasets owing to their greater number of layers. Moreover, DNNs are frequently employed as the dense layer in other deep-learning models [42]. DNN architectures are typically structured with a minimum of three fully connected layers, comprising an initial input layer, multiple intermediate hidden layers, and a final output layer. Within a fully connected network, individual neurons within a given layer are interconnected with all neurons from the preceding layer, facilitating comprehensive information exchange. The outputs generated by the hidden neurons are interpreted as a collection of distinct features derived from the input data specific to their respective layer. Successive layering within the network can be viewed as a process wherein features are abstracted and extracted at increasingly higher levels. Neurons situated in the nth layer are responsible for deriving features based on computations involving those originating from the (n-1)th layer [43]. The DNN architecture utilized in this study is depicted in Fig. 4. This architecture comprises 6 hidden layers. To enhance the training and generalization of the data in each network layer, batch normalization and dropout methods were implemented. Batch normalization standardizes the inputs of each layer using a fixed scale across the layer, ensuring that the outputs of the layers remain stable during the later stages of the learning process. Conversely, dropout deactivates a random set of neurons during each training step, aiding in preventing overfitting by reducing the model's reliance on each neuron. In this study, the dropout value is set to 0.2.

### 2.2 Convolutional neural network (CNN)

**Fig. 5:**
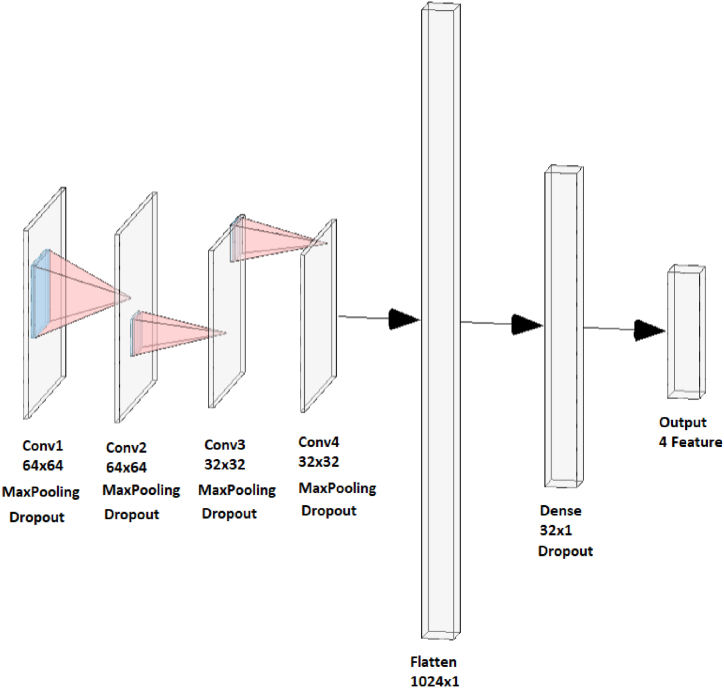
CNN model architecture.

CNN is a form of deep learning model extensively employed in tasks such as pattern recognition and image analysis. Its application has been prevalent within the domain of occupational health and safety, particularly within the context of construction sites [6]. It consists of several layers that can extract significant features from input data. CNNs are highly efficient in automatically extracting spatial information from data, especially two-dimensional image data. They are not as effective in processing one-dimensional data, such as physical or business data [44]. A common CNN architecture usually comprises a Convolutional layer, a Pooling layer, and a Fully connected layer. The Convolutional layer within a CNN employs filters to derive characteristics from the input data, with each feature necessitating an individual filter. The action of moving these filters across the input data is referred to as convolution. Within the CNN design, there are segments, each encompassing a convolutional layer with ReLU activation alongside a maximum max-pooling layer [44]. Furthermore, these filters ensure the preservation of distinct features extracted from individual sensor channels prior to their propagation to the subsequent network layers. The connections of the convolutional layers are established with the successive layers, in contrast to the fully interconnected structure seen in traditional neural network architectures. Also, for the purpose of classification, it is common practice to append a fully connected layer after the final block in order to consolidate the information gathered from all sensor channels. Subsequently, class probabilities are derived by incorporating a softmax layer at the conclusion of the network [43]. In certain cases, CNN may be more efficient than DNN due to its use of weight sharing and subsampling to decrease the number of weights and connections. The CNN architecture employed in this investigation is depicted in Fig. 5.

### 2.3 Long short-term memory (LSTM)

**Fig. 6:**
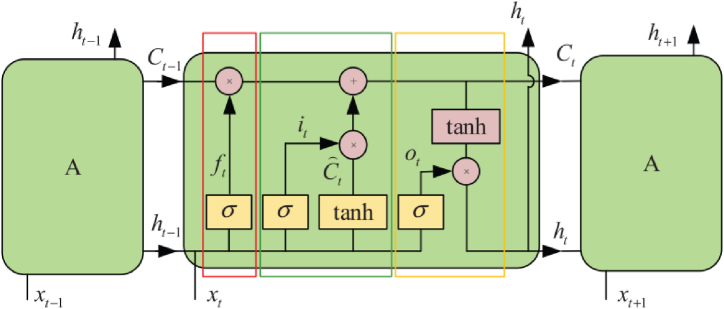
The basic structure of the LSTM cell [45].

**Fig. 7:**
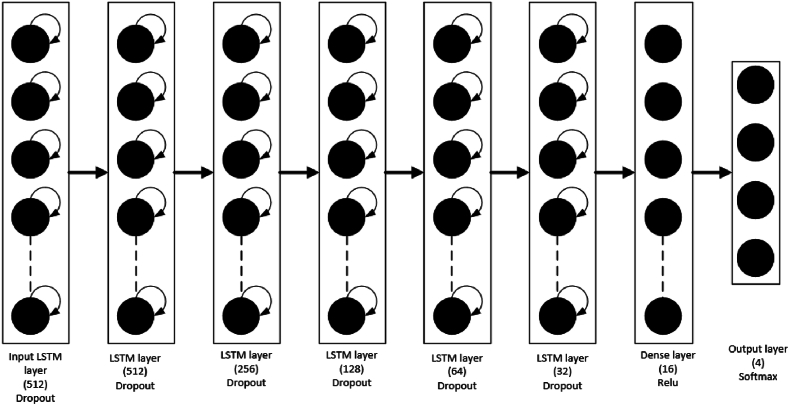
LSTM model architecture.

The LSTM model is a form of deep learning that excels in solving complex problems. Specifically, it is a widely successful variant of Recurrent Neural Networks (RNN), featuring layers with LSTM cells capable of storing information over time in internal memory. LSTM networks are adept at capturing temporal dependencies across various applications, including automatic translation, image captioning, and sensor or video-based event recognition [43,44]. Several studies have employed the LSTM model in the context of activity recognition [33,42,44]. LSTM cells are specifically engineered to retain and recall information over extended periods by retaining it within an internal memory. These cells are capable of updating, outputting, or deleting this internal state based on their inputs and the state from the preceding time step. LSTM cells are organized in hierarchical layers, akin to biological neurons. The information produced by each cell is conveyed to the succeeding cell within the corresponding stratum and subsequently to the subsequent stratum in the neural network. Upon reaching the final stratum, the information is forwarded to the dense and softmax strata to address the task of classification. The schematic in Fig. 6 depicts the fundamental composition of an LSTM cell, which comprises the forgetting gate (red rectangular part), input gate (green rectangular part), and output gate (yellow rectangular part) [45]. The architecture of the LSTM model used in this study is depicted in Fig. 7. It comprises 6 LSTM layers, with each layer containing a specific number of LSTM cells. Following the LSTM layers, a dense layer utilizing the Relu activation function and an output layer employing the softmax activation function were constructed.

In the field of literature studies, various deep learning techniques have been employed for the purpose of recognizing human activities and enhancing occupational health and safety protocols within construction sites as well as other industrial settings [6,31,[33], [34], [35],[46], [47], [48], [49]]. In this study, the primary aim is to determine the most suitable model through the analysis of literature-derived data using various deep learning methods and diverse window and overlap ratios. To achieve this objective, the analysis results have been assessed using a range of evaluation metrics. Various metrics such as Accuracy, F1-score, and Loss values are considered in the evaluation process. The metrics Accuracy and F1-score are determined through the application of specific formulae denoted by Equations (1), (2). Within the realm of deep learning classification tasks, Loss values are computed utilizing Equation (5). This metric is crucial for assessing the model's predictions against the ground truth. It quantifies the disparity between the model's predictions and the actual values, with a lower Loss value signifying a higher level of proximity between the predicted and actual values.

(1) $$Accuracy=\frac{TP+TN}{TP+FP+TN+FN}$$

(2) $$Precision=\frac{TP}{TP+FP}$$

(3) $$Recall=\frac{TP}{TP+FN}$$

(4) $$F1\;Score=\frac{2*Precision*Recall}{Precision+Recall}$$

(5) Loss=−∑(y∗log(p))

where y is the true value and p is the predicted value of the model.

## 3 Results and discussion

**Fig. 8:**
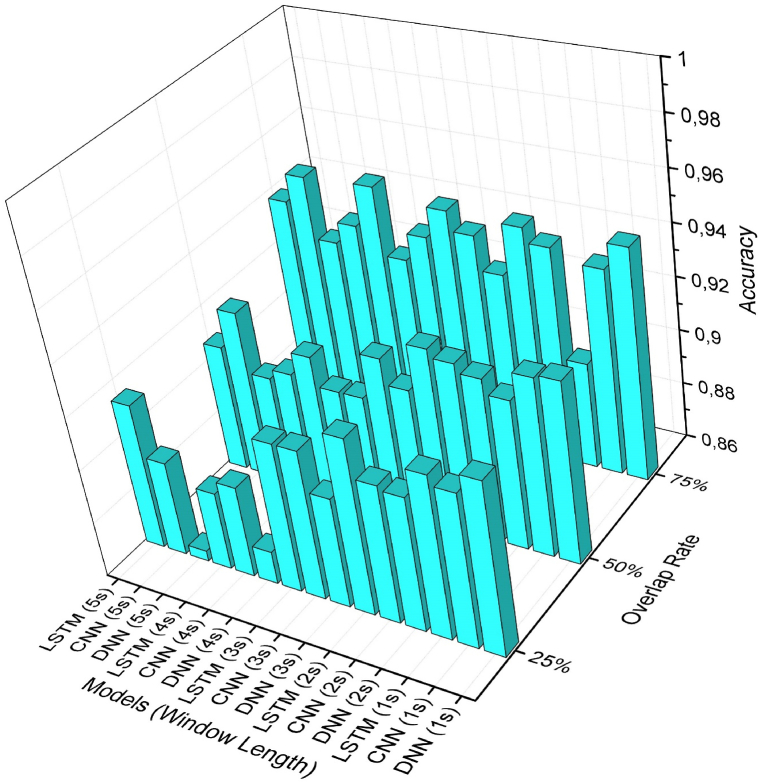
Accuracy values of the analysis results.

This study utilized deep learning methods to analyze acceleration, gyroscope, and pressure data, aiming to predict the working conditions of workers on three different types of scaffolds as well as when they are on the ground. The analysis results allow us to predict whether a worker is on a scaffold or on the ground, and if on a scaffold, which type it is (mobile scaffold, horse scaffold, or ladder). This analysis has also facilitated an evaluation of working at heights. Additionally, it has been sought to identify the most suitable model by creating data sets from different window sizes and overlap rates for each deep learning method. 15 data sets were generated for each model, and a total of 45 analyses were conducted for the purpose of identifying the most appropriate model for working at heights. The analysis results revealed the most suitable model, with Fig. 8 displaying the accuracy values obtained. The models with a 75 % overlap rate generally exhibit higher accuracy compared to models with different overlap rates. The window lengths vary for each model. Regarding the accuracy of the deep learning models, the DNN model demonstrates a higher accuracy rate with a 1-s window size, while the CNN model shows a higher accuracy rate with other window sizes. To effectively evaluate the prediction success of these models, it is important to consider the loss and f1 score values in addition to accuracy.

**Fig. 9:**
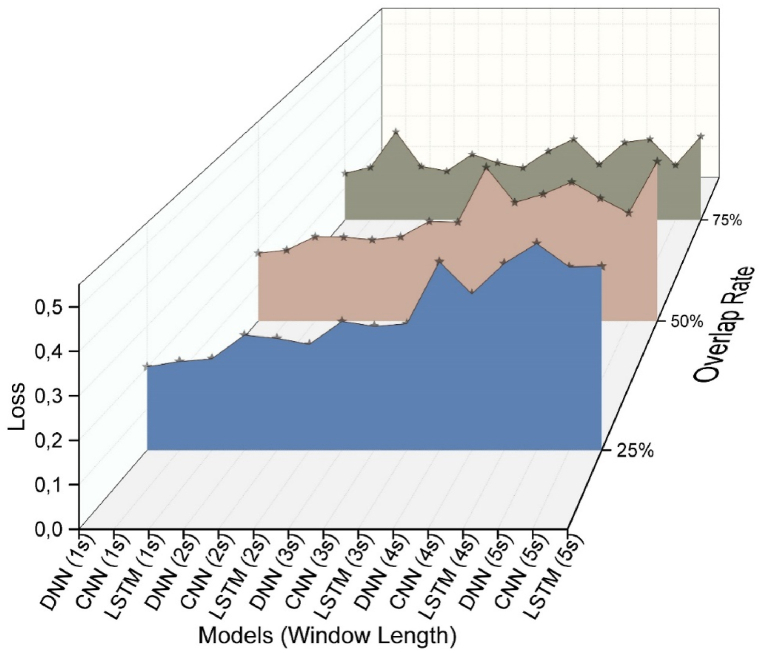
Loss values of the analysis results.

**Fig. 10:**
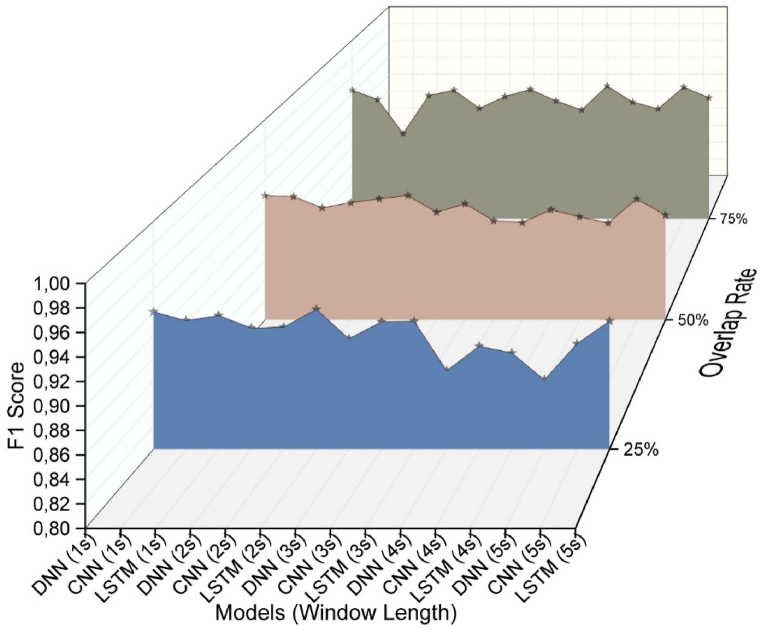
F1 score values of the analysis results.

When analyzing the F1 score and Loss values, it is evident that the highest prediction success is achieved with 75 % overlap rates (Fig. 9, Fig. 10). Upon reviewing the prediction results of LSTM models, it is apparent that they exhibit the highest level of loss. Additionally, CNN and DNN models demonstrate better prediction results compared to the LSTM model.

In the above figures, it has been observed that deep learning analysis yields higher accuracy rates at 75 % overlap rates. Therefore, Table 2 presents the analysis results at a 75 % overlap rate. It is evident that the CNN model generally outperforms the DNN model. The most successful models were the DNN model with a 1s window size, achieving a prediction success of 94.6 %, and the CNN model with a 5s window size, achieving a prediction success of 94.9 %.

**Table 2:** Results of the 75 % overlap rate deep learning models.

Model	Win Length	Accuracy	Loss	F1-score
DNN	**1s**	**0,9460**	**0,1445**	**0,9441**
	2s	0,9401	0,1660	0,9380
	3s	0,9389	0,1770	0,9371
	4s	0,9239	0,2503	0,9219
	5s	0,9250	0,2498	0,9232
CNN	1s	0,9360	0,1627	0,9336
	2s	0,9457	0,1510	0,9441
	3s	0,9465	0,1618	0,9449
	4s	0,9503	0,1718	0,9488
	**5s**	**0,9490**	**0,1696**	**0,9475**
LSTM	1s	0,8987	0,2724	0,8955
	2s	0,9259	0,2030	0,9238
	3s	0,9345	0,2130	0,9321
	4s	0,9334	0,2397	0,9309
	5s	0,9377	0,2585	0,9357

**Fig. 11:**
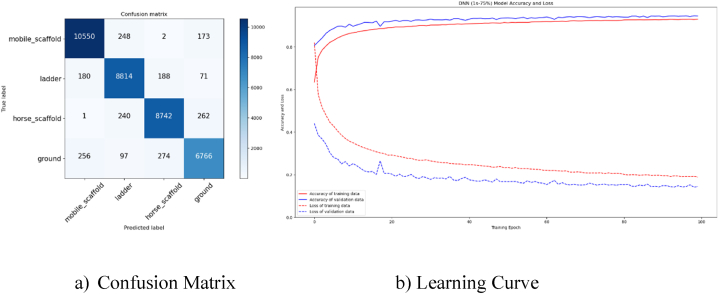
Confusion Matrix and Learning Curve for 1s (75 %) DNN model.

Upon evaluating the models with the highest accuracy rates individually, it was found that the DNN model, analyzed with a 1-s window size and a 75 % overlap rate, achieved a prediction accuracy of approximately 95 % for scaffold type. Fig. 11(a) presents the confusion matrix of this analysis, while Fig. 11(b) displays the learning curve graph. The confusion matrix indicates that the model more accurately predicts the worker's working situations, including mobile scaffold, horse scaffold, ladder, and ground. Evaluating a model's performance can be effectively done by plotting its learning curve. These curves illustrate the relationship between accuracy and the quantity of training examples. By graphing the training and cross-validation scores for a particular model across different training dimensions, we can observe how the scores change as the number of training examples increases. Additionally, these curves can help identify whether the model suffers from bias or variance. A significant margin between training and validation accuracy could indicate overfitting of the model.

In the evaluation of the confusion matrix, the DNN model correctly predicted 10550 out of 10973 mobile scaffold data. However, it had some confusion with the ladder class. Out of 9253 ladder data tested, 8814 were accurately predicted, but there were some instances of confusion with the mobile scaffold and horse scaffold categories. Lastly, out of 9245 horse scaffold data, 8742 were correctly predicted. The data set was also mixed up with ladder and ground information. Amongst the 7393 ground data points that were analyzed, 6766 were accurately forecasted, often mistaken for mobile and horse scaffold data. However, based on the learning curve graph, we can infer that the DNN model is not prone to overfitting, as there is only a minimal difference between the training and validation lines.

**Fig. 12:**
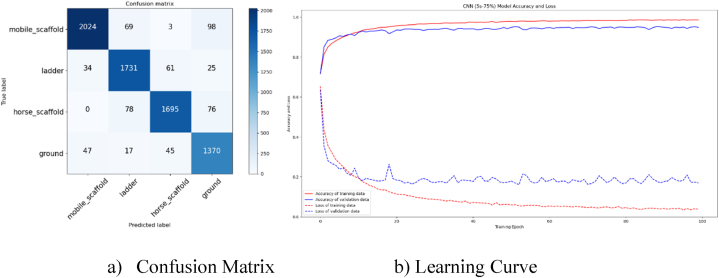
Confusion Matrix and Learning Curve for 5s (75 %) CNN model.

The CNN model was assessed using a 5-s window size and a 75 % overlap rate, achieving an accuracy of approximately 95 % in predicting scaffold types. Fig. 12(a) illustrates the confusion matrix for this analysis, while Fig. 12(b) depicts the learning curve graph. According to the confusion matrix, 92 % of the 2194 mobile scaffold data tested with the CNN model were correctly predicted, with 4 % misclassified as ground. For the 1851 ladder data tested, 93 % were accurately predicted. The analysis indicates that there is some confusion between the ladder and horse scaffold categories. Among the 1849 instances of horse scaffold data, 92 % were accurately classified, but 4 % of these instances were mistakenly identified as ladder or ground data. Similarly, 93 % of the 1479 ground data samples were correctly predicted, with 3 % being misclassified as mobile or horse scaffold data. Despite the greater separation between the training and validation lines on the learning curve graph compared to the DNN model, it is apparent that the CNN model is not overfitting.

In this study, a proposed method aims to determine whether construction site workers are working on scaffolding or on the ground. If the workers are on scaffolding, the method seeks to identify the specific scaffolding they are using. The study evaluates various window sizes and overlap ratios during model analysis to establish the optimal settings for the model. Based on the obtained results, the study aims to assess whether the workers are working at height and if they have taken the necessary precautions for working at that height. The dataset utilized in this study was sourced from Choo et al. (2023) [2]. The study involved determining height by employing pressure data and Kalman and complementary filters, resulting in significant computational overhead. Notably, the analysis focused solely on ascertaining whether the workers were operating at height. Furthermore, deep learning techniques were applied to forecast the specific scaffold on which they worked. The process reduced the crowd and employed a quicker method to determine whether the worker was working at height or not, with an accuracy rate of 95 %. In addition to pressure data, this study utilized accelerometer and gyroscope data for height estimation, considering the unique movements of workers on each scaffold. Furthermore, by gathering this data, future analyses such as activity recognition and productivity calculation can be performed. Anjum et al. (2022) [6], sought to measure the working height of workers using a ladder in their study. They employed a deep learning-based computer vision method to achieve a height estimation accuracy of around 85 %. The study effectively estimated the working heights of the workers using just one sensor. In their review research, Khan et al. (2023) [8], highlighted falls from height as a critical occupational health and safety issue in construction sites and stressed the need to integrate advanced construction technologies for prevention. However, they found the current technologies to be inadequate. Our study proposes a deep learning model that can accurately predict whether a worker is working at height using data from a single sensor. This innovation is expected to facilitate easier control for occupational health and safety experts at construction sites and help reduce FFH accidents. In their review article, Newaz et al. (2022) [24], provided an extensive evaluation delving into the impact of FFH technologies and their suitability on construction sites. Drawing from their research findings, a total of 7 FFH technologies were pinpointed to delineate their role in forecasting, averting, and alleviating FFH hazards. These encompass (1) safety risk assessment and propagation, (2) real-time sensing and monitoring, (3) automated prevention through design, (4) ontology and knowledge modeling, (5) virtual reality for FFH training, (6) personal fall arrest systems, and (7) collective fall protection systems. The present study aims to enhance the anticipation and prevention of FFH risks among construction workers by integrating real-time sensing and monitoring technology into the realm of automated prediction of workers operating at elevated levels.

This proposed method can be employed to establish a worker safety monitoring system on construction sites. For example, a system that automatically classifies workers based on their working environment could send alerts to employers or safety teams in hazardous situations. This capability is especially critical for workers on ladders or scaffolding, where the risk of falls is prevalent. Consequently, occupational safety teams can intervene swiftly to prevent potential accidents. Furthermore, the data collected can be routinely analyzed to pinpoint potential hazards on construction sites, enabling the implementation of proactive safety measures. Additionally, these insights can be integrated into occupational safety training programs. By analyzing worker behavior in relation to the findings, it becomes possible to identify which working environments carry greater risks, thereby allowing for the development of targeted training programs that focus on these specific areas. Real-time monitoring plays a crucial role in identifying issues such as improper equipment usage and deviations from established procedures. For example, if a worker is detected operating at excessive heights, this indicates a potential safety concern. Consequently, an automatic warning can be issued, prompting the worker to use the appropriate occupational health and safety equipment. By creating a digital twin of a construction site that models the working environment and integrates deep learning analytics, we can effectively identify potential vulnerabilities. This method enables us to simulate the heights at which workers operate, providing essential insights into safety risks.

## 4 Conclusion

The construction industry is currently undergoing a technological revolution, largely due to the integration of IoT and sensor technologies. In addition to these, artificial intelligence methods are commonly employed in the literature to monitor and analyze workers in the realm of occupational health and safety. These advanced tools serve various purposes, including identifying worker activities through sensors, automatically calculating worker productivity, assessing occupational safety risks, determining workers' activities at heights, and detecting worker fatigue. The primary objective of this study is to utilize accelerometer, gyroscope, and pressure data to assess the scaffold on which a worker is operating and consequently establish the precariousness associated with working at elevated heights.

In the construction industry, assessing the safety risks faced by workers traditionally relies on labor-intensive manual observation and the expertise of safety professionals. However, this approach is time-consuming. To improve efficiency, a proactive framework for assessing labor safety risks is essential. This framework should also address the issue of falls from height (FFH). As part of our study, we aim to analyze the scaffolds where workers are stationed using data gathered from the workers themselves to gain insights into FFH risks. According to FFH evaluations in many developed countries such as Korea, the USA, and the UK, mobile scaffold and ladder work is considered as working at height. In contrast, ground and horse scaffold work is not. In this study, based on prediction results obtained from sensors, it can be inferred that working at height conditions should be applied when it is determined that work is being conducted automatically on a scaffold that is categorized as working at height.

In this research, it has been employed deep learning methods to analyze acceleration, gyroscope, and pressure data gathered from construction workers. The aim of the study has predicted the working conditions of these workers across three different types of scaffolding and on the ground. The analysis enabled us to determine whether a worker was on scaffolding or on the ground, and if on scaffolding, to identify the specific type—namely, mobile scaffolding, horse scaffolding, or ladder. It has been evaluated each deep learning model based on various window sizes and overlap ratios, resulting in a total of 45 distinct analyses. The findings demonstrated that the CNN model, configured with a 75 % overlap rate and a 5-s window size, achieved accuracy, loss, and F1 score values of 0.9490, 0.1696, and 0.9475, respectively. Similarly, the DNN model with the same overlap rate and a 1-s window size explained accuracy, loss, and F1 score values of 0.9460, 0.1445, and 0.9441, respectively. Among the deep learning models assessed, both the CNN (5-s window size with a 75 % overlap rate) and the DNN (1-s window size with a 75 % overlap rate) exhibited exceptional prediction accuracy of approximately 95 % when identifying the locations of workers. This remarkable level of accuracy underscores the potential for integrating these systems into safety monitoring installations on construction sites.

The findings of this study illustrate the potential for enhancing construction management, particularly in complex and large-scale projects, through the integration of sensors in the work environment. This innovative technology is poised to make a significant impact on occupational health and safety, especially within construction sites. The model developed in this research is capable of real-time analysis of workers' operational statuses. In hazardous areas, this functionality enables prompt interventions, thereby improving occupational safety and reducing the risk of accidents. Additionally, this study enhances prediction accuracy by leveraging data from a variety of sensors, including accelerometers, gyroscopes, and barometers, rather than relying solely on a single sensor. Achieving high-accuracy predictions for preventing falls from heights is expected to provide considerable benefits to the construction industry, particularly regarding worker safety and minimizing project downtime. Nevertheless, it is important to acknowledge that the research has its limitations. While our model exhibits high accuracy rates, there may still be instances of false positive and false negative predictions. For example, a worker could be mistakenly assessed as being ‘in danger’ based on movement data, resulting in unnecessary alarms. Additionally, environmental factors on construction sites—such as dust, humidity, temperature fluctuations, and impacts—can interfere with the sensors' functionality. Moreover, ensuring that workers consistently wear the sensors presents practical challenges. The sensitivity and calibration of the employed sensors significantly influence the model's performance. Subpar or improperly positioned sensors can diminish data accuracy and adversely affect prediction outcomes. Continuous monitoring of workers' movements may also lead to privacy concerns, raising both ethical and legal implications. To mitigate these issues, strategies like anonymization and the analysis of only the necessary data can be implemented. Furthermore, expanding the dataset with data collected under varying conditions can enhance the diversity and scope of the training information. To tackle these challenges, it is essential to develop smaller, more robust sensors and conduct regular maintenance. These advancements can support the seamless integration of technology within construction sites. Future research can expand on the development of sensors by implementing them in real construction site environments and involving numerous workers. Furthermore, the scope of occupational health and safety procedures can be broadened by gathering additional data and conducting analyses to assess whether appropriate protective measures are being taken based on height conditions. The accuracy of the proposed model can be assessed and refined by gathering data across various environmental conditions and from more complex workplaces. Future research priorities could include enhancing the durability of sensors, developing devices with lower energy consumption, and exploring innovative technologies capable of collecting more sensitive data. Furthermore, integrating motion data with other types of information, such as GPS and RFID, could lead to more comprehensive analyses in the future. This data integration allows for more precise monitoring of workers' locations, heights, and behaviors. Conducting long-term analyses of the collected data can help identify recurring risk factors in the work environment and support the development of proactive measures. Future research can leverage workers' movement data not only to enhance safety but also to conduct ergonomic assessments and identify potential risks to the musculoskeletal system. With ongoing advancements in technology, the efficacy of predictive models can be further improved by incorporating various artificial intelligence methodologies. To facilitate real-time analysis for employers and technical staff, it is essential to enhance the integration of software and hardware. Specifically, the proposed model could benefit from being integrated with mobile applications or cloud-based solutions. Moreover, the methods developed in this study can be adapted for use in sectors beyond construction and tested across different working environments. Such adaptations could increase the system's flexibility and expand its overall applicability.

## Data availability statement

All data generated or analyzed during this study are included in this published article.

## Code availability

Not applicable.

## Funding

The authors declare no funding for this research.

## Declaration of competing interest

The authors declare that they have no known competing financial interests or personal relationships that could have appeared to influence the work reported in this paper.
